# Keeping the lights On or Off: tracking the progress of access to electricity for sustainable development in Nigeria

**DOI:** 10.1007/s10708-022-10689-2

**Published:** 2022-07-03

**Authors:** Oluwafisayo Alabi, Aisha Abubakar, Astrid Werkmeister, Suki Dauda Sule

**Affiliations:** 1 Centre for Energy Policy, School of Government and Public Policy, University of Strathclyde, McCance Building, 16 Richmond Street, Glasgow, G1 1XQ UK; 2 Department of Architecture, Faculty of Engineering, University of Strathclyde, Royal College Building 204 George Street, Glasgow, G1 1XW UK; 3 Department of Electrical and Electronic Engineering, Faculty of Engineering, University of Strathclyde, Royal College Building 204 George Street, Glasgow, G1 1XW UK

**Keywords:** Electricity access, Satellite Night lights (SNLs), Satellite imagery, Sustainable development tracking

## Abstract

This paper is focussed on employing satellite night lights (SNLs) to investigate access to electricity across the geographical regions in Nigeria. Specifically, we explore how SNLs interact with human and socioeconomic development indicators (population, poverty, and household consumption) to demonstrate the implications of slow and/or delayed progress in closing the electricity access gap in Nigeria. Our findings suggest that minimal progress has been made and there remains significant evidence of disproportionate spread of electricity across the country with most of the electricity visibility concentrated in the Southern regions, state capitals and industrial centres. Crucially, policy challenges and trade offs emerge. On one hand, is the need to address the long-standing issue of stranded and underutilised assets around power generation, transmission, and distribution and how these balance (or not) against additional and new capacity to enable sufficient, reliable and sustained electricity supply. On the other hand, is the challenge of ensuring that closing the access to electricity gap in Nigeria is done in a way that is just, fair, and equitable, with no part of society becoming worse-off or excluded.

## Introduction

At the United Nations Sustainable Development Goals (SDGs) summit in 2019, world leaders marked 2020–2030 as a crucial ‘decade of action’ to achieve sustainable development.^1^ Several recommendations were made on the need for more ambitious actions around mobilising financing, enhancing national implementation, and strengthening institutions, with the overarching plan of leaving no one behind. Underlying these actions are various SDGs tracking reports that suggest that some advancements and progress have been made across the globe, but a long road is still ahead (IRENA, 2020). Moreover, a more complex challenge now arises around the slow speed or pace of progress relative to the required scale of advancement to achieve a sustainable future for all, and the potential of undoing any progress made owing to this. The challenge is at further risk due to the impacts of the Covid-19 pandemic and ongoing and potentially long term economic and societal recovery and resilience needed, especially in developing countries (IRENA, 2020).

Focussing attention on SDG 7^2^: particularly the context of access to electricity, its attainment is looking less promising. Currently, it is estimated that around 789 million people (out of a total of 1.14 billion),^3^ predominantly in Sub-Saharan Africa, are living without access to electricity, and hundreds of millions more only have access to very limited or unreliable electricity (UN, 2020). In Nigeria, issues around the disproportionate spread of electricity, interrupted electricity supply, and power outages across unserved and underserved populations remain the major factors hindering human and economic development (NBS, 2020; Sarkodie & Adams, 2020). Nonetheless, a range of Nigerian national policies, power sector recovery programmes and strategies: 4 have been developed to address the different challenges around the electricity sector through pathways involving advancement of on-grid and off-grid solutions driven by a range of renewable energy solutions to accelerate the transition and delivery of access to electricity for all by 2030. For instance, the Nigerian sustainable energy development initiative action agenda sets out the following targets^5^:

1. To increase electricity access from the aggregate level of 40% (urban 65% and rural 28%) in 2015 to 75% (urban (65%) and rural (60%) by 2020.
2. By 2030, the population living without energy access should drop from 60% in 2015 to about 10%
3. Increase electricity generation from the present grid supply of 5,000 MW in 2015 to at least 32,000 MW by 2030
4. Increase overall supply of electricity in the country to 23.5GW and 45 GW by 2020 and 2030 respectively).
5. Increase On-grid supply from 26% (2016) to 48% (2020) and 70% (2030)
6. Reduce the use of self-generated power from 74% to about 48% and 18% in 2020 and 2030 respectively.

For Nigeria to effectively track any of the sustainable energy development targets, national-oriented and territorial continuous and consistent monitoring as well as assessments, is required, to provide a clearer picture on the progress made (or not). Such insights are imperative, if both national and international decision makers are to better understand the scale of the challenge and in turn pre-identify and/or identify the type of pathways, strategies and incentives that can promote and accelerate their achievement. However, a critical and long-standing challenge, for Nigeria like many other developing countries, is the issue of inadequate and unreliable data for tracking and assessing the progress of sustainable development (Bennett & Smith, 2017; Fayomi et al., 2019; Kraak et al, 2018; Mentis et al., 2015).

In this paper, we make a first attempt, to the best of our knowledge, to show how satellite data and imagery, particularly Satellite Night Lights (SNLs) can be used to address the data limitations and gaps arounds tracking the progress of electricity access in Nigeria. In addition, we also make a further contribution by addressing a gap in the literature of demonstrating the extent to which the state and progress of electricity access impacts and interacts with human and socio-economic development indicators (e.g., population, poverty and households’ expenditure and consumption) using SNLs. The focus on such development indicators, are identified as key to ensuring SDGs in any one national and at the global level is delivered and achieved in an equitable and fair way for all (UN, 2021, 2020).

Crucially, our motivation for employing SNLs is underpinned on the fact that satellite data and imagery are internationally recognised and accepted data sources for monitoring sustainable development indicators (Sustainable Development Solutions Network SDSN, 2015). According to Andries et al. (2022), there is a potential to populate around 108 sustainable development indicators by using Earth observation data only. Satellites, in general, depending on their mission, carry different payloads -in the case of Earth observation, they have different sensors on board, which can measure different properties and element of the Earth’s oceans, land, and atmosphere. In this work, we demonstrate how geographical SNLs and ground-based data, can be used to develop, improve, and strengthen national systems for monitoring, tracking, and assessing the progress of sustainable development goals, using the illustrative case of SDG 7, with particular focus on access to electricity.

The objectives of this work are to address the following questions:

1. What is the share of electricity generation in Nigeria and who has access to it?
2. How much progress has been made in closing the electricity access gap across the geographical regions in Nigeria?
3. How is household spending and consumption impacted by insufficient electricity supply?

The remainder of the paper is structured as follows: Sect. 2 provides a literature review on the application of SNLs data to consider various questions on access to electricity in relations to sustainable development indicators. Here we lay emphasis on SDG 7. Section 3 provides a description of the data and method and the analytical approach employed. Section 4 discusses the results and highlights the narrative emerging from the analysis. Finally, the paper concludes and proffers policy recommendations and potential future research directions in Sect. 5.

## Literature on access to electricity for sustainable development and Satellite Night Lights (SNLs)

There is already an extensive and growing literature identifying the opportunities and challenges around SDG7 in Nigeria with focus on the element of electricity access using various techniques, methods, and analysis. For example, several studies argue that the opportunities around improving electricity access lies in exploiting and increasing development in renewable energy resources and technologies to diversify the energy mix and reduce reliance on fossil fuel energy sources (Oyedepo, 2014; Onabote et al., 2021; Modibbo et al., 2021). Other studies suggest that the barriers and challenges around the lack of accelerated investment and financial incentives as well as existing infrastructure failures must be addressed, if renewable energy resources are to play a sufficient role in enabling access to electricity. (Adenikinju, 2005; Nwokoye, 2008; Utazi & Ujam, 2014).Attention has also been given to the challenges, concerns and issues on poor policy formulations, implementations, and performance, for supporting the delivery of energy security, justice, and ultimately enabling access to electricity for all.(Emodi et al., 2015; Hamza et al., 2020; Monyei et al., 2018; Müller et al., 2021).

However, we argue that while important issues and questions have been addressed around electricity access in Nigeria, there is a missing debate and discourse around tracking and monitoring the progress of SDG 7. Müller et al. (2021) notes that to combat energy poverty and make good on SDG 7, Africa is challenged by a lack of comparative studies to track such transition, and this relates to an epistemic and institutional mismatch in terms of key and tailored policies. Our contribution gains distinction from existing studies in two ways. First, we address a key gap around the data limitations and constraints of tracking access to electricity by employing Earth observations in the form of satellite data and imagery; SNLs. Secondly, we consider how the current state of access to electricity affects and impacts human development and wellbeing (population, poverty, and household expenditure)  across the geographical  regions in Nigeria.

SNLs and satellite imagery in general are major data sources, and a unique and powerful tool that can be employed to detect, spatially map, estimate and monitor various dynamics of human activity and development (Doll et al., 2006; Levin & Duke, 2012; Ustaoglu et al., 2021). For instance, SNLs have been applied to consider the state of urban development, economic growth, electricity consumption and population distribution across different countries and regions (for example see Amaral et al., 2005; Elvidge et al., 2014; Sutton et al., 2007; Townsend & Bruce, 2010). Some studies have used SNLs to examine the impacts of carbon emissions and footprints (Milesi et al., 2003) and the populations at risk of natural and environmental disasters such as radiation and volcanic eruptions (Dobson et al., 2000; Kohiyama et al., 2004). Focusing on monitoring societal welfare using SNLs, Cova et al (2004), Zhang and Seto (2011), investigate the relationship between quality of life, infrastructural development, sustained economic activity, and wealth as key components of human development.

More recently, the application of SNLs has also extended to considering the composition and transition to the SDGs by measuring increases in SNLs overtime to capture the patterns and trends of electricity visibility and level of electricity consumption. For example, Townsend and Bruce (2010) use SNLs to measure regional electricity consumption and population distribution in Austria. Stokes and Seto (2019) explore how fusing multi-temporal population and land data with SNLs data, derived from the Suomi-NPP VIIRS Day Night Band, can provide better understanding of urban infrastructural transitions to create a typology of urban development processes. The study tracks rural electrification by identifying growing informal settlements with inadequate infrastructure. Other studies use SNLs mapping and imagery to investigate the problem of energy poverty. For instance, Falchetta et al (2020) apply SNLs to examine the progress and effectiveness of electrification in Africa. A core outcome of the study points to the fact that the level and state of electricity access is significantly dependent on sufficient consumption and reliability of supply.

Among previous studies, SNLs imagery has also been employed to assess inequality and poverty levels. Wang et al. (2012) use SNLs imagery and spatial data to study the extent and nature of gaps in socio-economic development levels across 31 regions in China. While Mveyange (2015) sought to augment lack of income data to study inequality in African regions through SNLs mapping. Doll and Pachauri (2010) focus on how spatial dynamics and dimensions can be used to trace and estimate populations without access to electricity in Sub-Saharan Africa. These authors find that lack of access to electricity among the rural population is attributable to low density and high dispersion of the population as well as income levels. Bennett and Smith (2017) and Zhou et al. (2019) provide a comprehensive review of the advances in data processing and multitemporal analysis in improving the interpretation of SNLs imagery in understanding socioeconomic dynamics.

We contribute to the growing literature by proposing how SNLs imagery may be used to investigate the progress on access to electricity in Nigeria where there has been limited application of spatial information in supporting the estimation, tracking, and monitoring of the phenomenon for sustainable development. Here, we optimise and focus on three interactive elements of access (see Penchansky & Thomas, 1981) as it applies to the distribution and use of electricity: (1) accessibility, which is tied to availability, (2) affordability, and (3) adequacy. Our contribution is timely, as national policy makers are faced with the complexity of understanding how to enable and deliver sustainable development in a way that is just and fair across all levels of society. Asset and resource development is only effective when society has equitable access, this is no different for energy, which is key to boosting economies (Jana, 2016; Thomas & Amadei, 2010; UN-Habitat, 2020). Moreover, limited access to energy resources can significantly affect societal wellbeing and in turn trigger increased poverty levels (Eneh, 2021; Habitat for Humanity, 2021). Hence, reconsidering or resetting SDGs including SDG7 as a societal challenge is necessary, and without focussed and timely policies to understand the social cost vs benefits, consistent access to electricity may be hampered and unsustainable for a large part of the population.

## Data and research methods

**Fig. 1:**
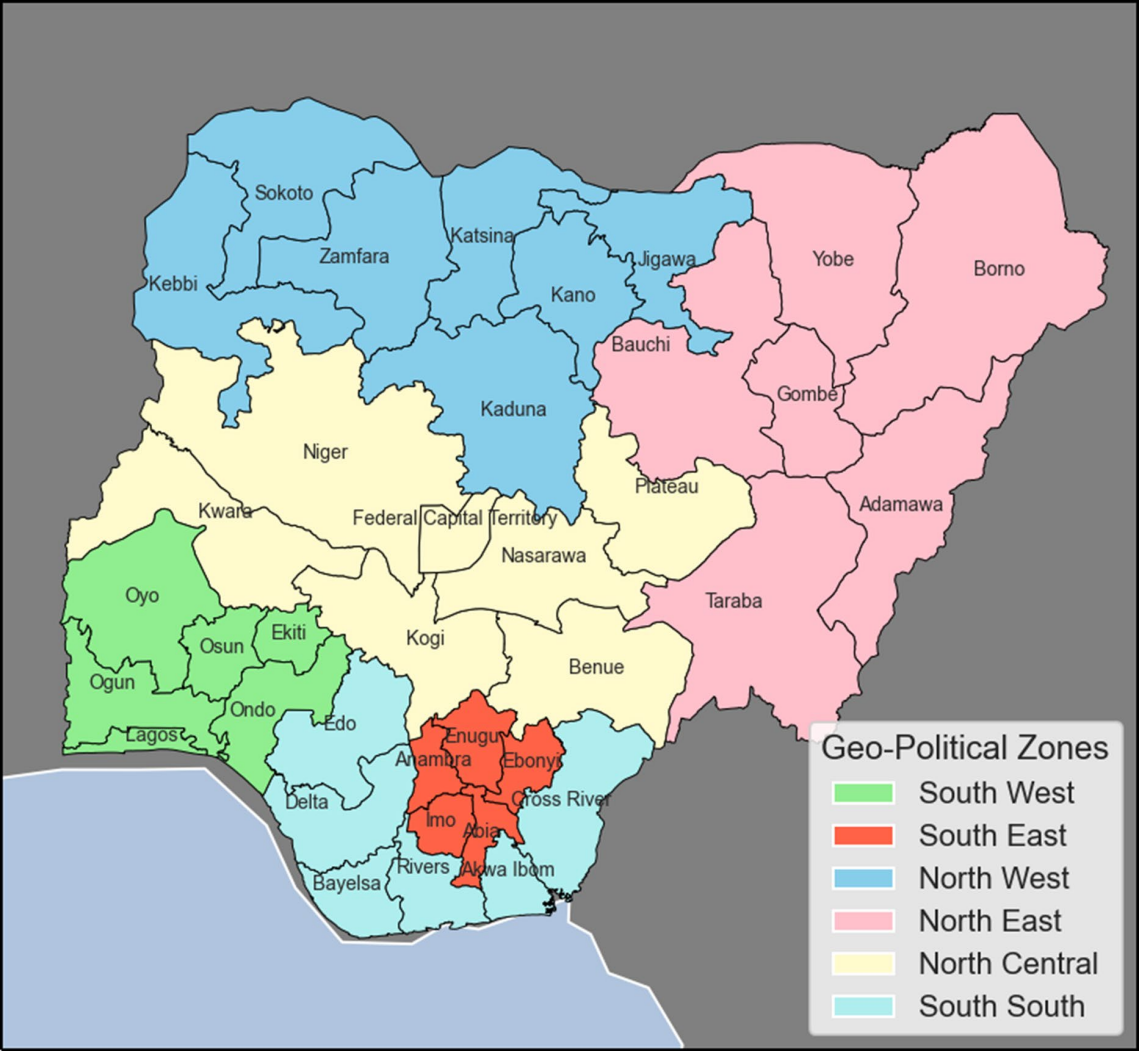
The geographical zones in Nigeria

Our analytical approach involves three main steps: First, we begin by describing the structure, components, and distribution network of electricity supply within regions and across the major geographical zones in Nigeria. Currently, Nigeria has six geographical zones (see Fig. 1) that categorise the 36 states based on common ethnicities, culture, and history, and are diverse in their physical and social geography (see Eze et al., 2014). These include (1) South West, (2) South East, (3) North West, (4) North East, (5) North Central, And (6) South South zones (Fig. 1). We also capture other relevant socio-economic and developmental data that can help provide insight on the access to electricity in Nigeria; these are discussed in Sect. 3.1. For the second stage, we use the SNLs data for selected years/periods (2016–2019) to illustrate a visual approximation of electricity availability. The methodology employed in this regard is outlined in Sect. 3.2. In the third stage, the interaction between step one and two is determined by identifying patterns in the areas/regions with highest and lowest share of electricity availability based on the SNLs for 2019 as discussed in Sect. 3.3.

### Ground data (non-satellite data)

**Fig. 2:**
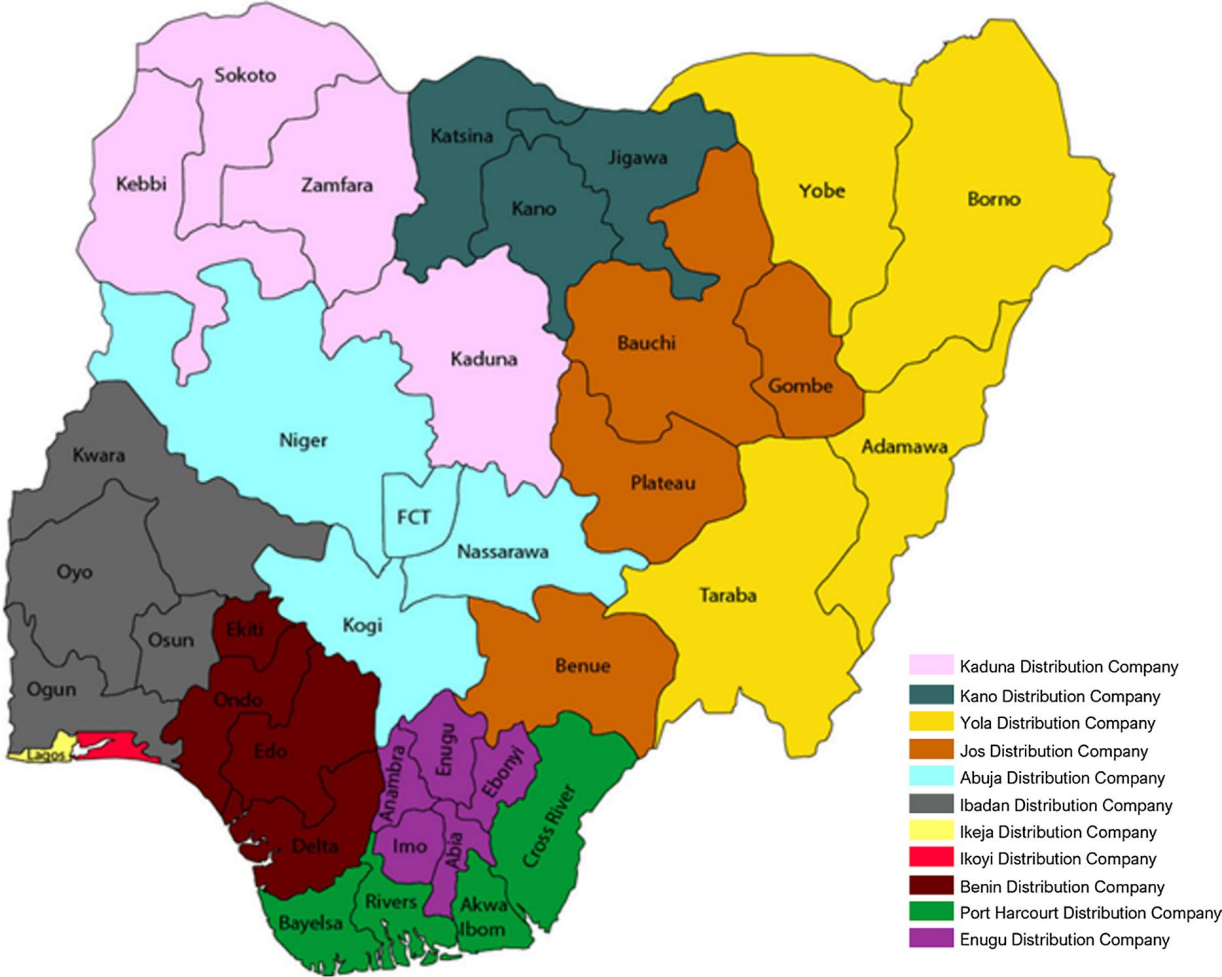
Electricity distribution company coverage areas in Nigeria

**Fig. 3:**
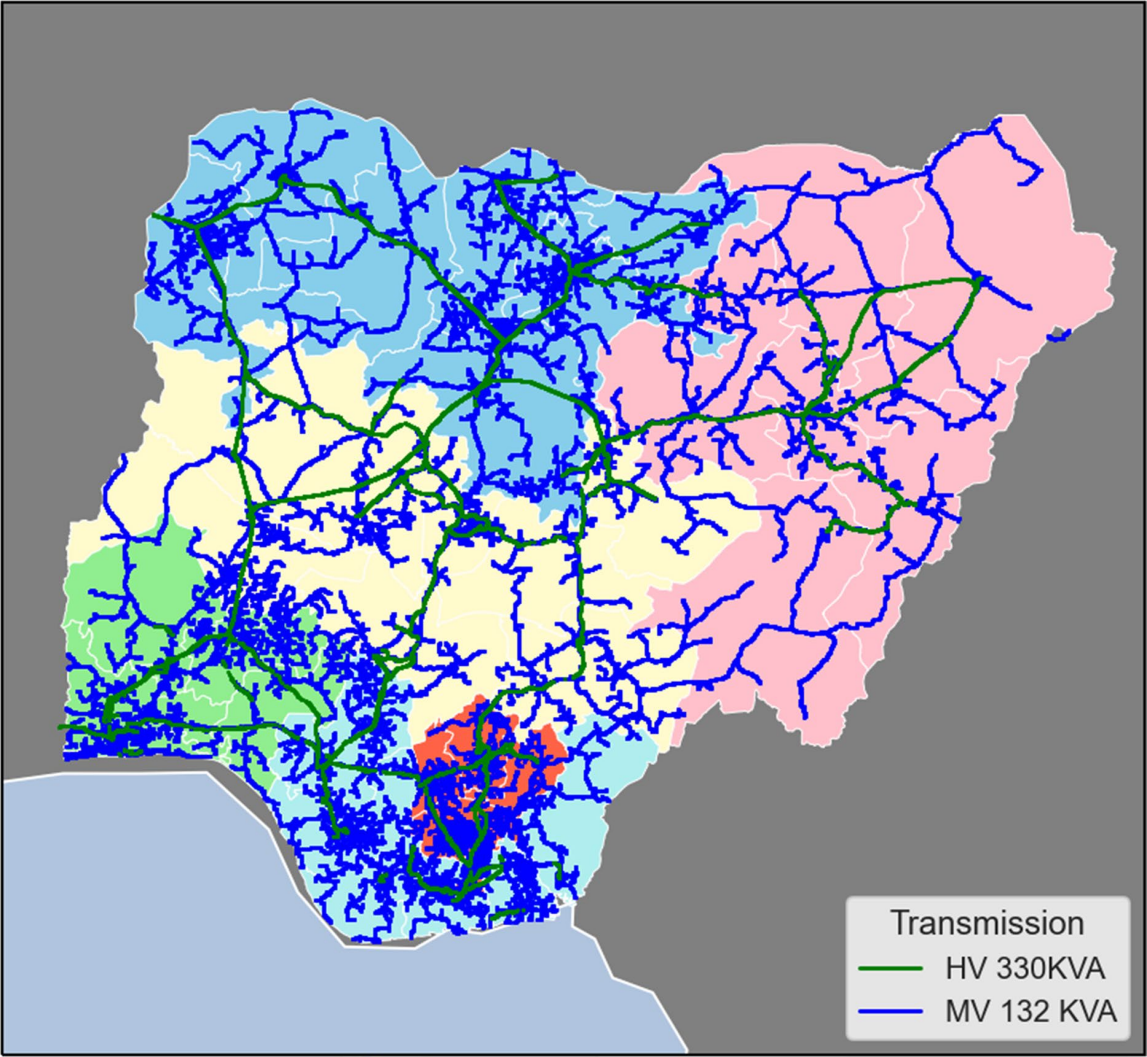
High and Medium Voltage Transmission Line Network in Nigeria

**Fig. 4:**
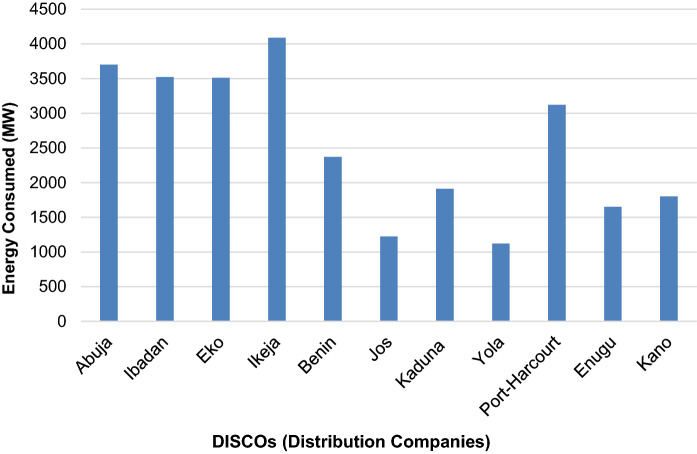
Total Energy Consumed by DISCOs for January-December 2019

The ground data on electricity supply is derived from actual electricity consumption data from the distribution companies (DISCOs). There are currently 11 DISCOs in total in Nigeria that supply electricity to all end users (residential, commercial and others) (NERC, 2020). Load shedding is also implemented by the DISCOs in the distribution of electricity to end users across the six geographical zones. Figure 2 maps each DISCO to the regions of electricity distribution coverage, while Fig. 3 shows the actual physical energy transmission across the country. The actual electricity consumption data (ground data) from the DISCOs is obtained for the whole year 2019 and is plotted in bar charts, shown in Fig. 4. We observe that the electricity consumption data for the years 2016 to 2018 to be somewhat incomplete. For instance, the monthly consumption for 2018 was unavailable and there was no energy consumption data for 2016, while consumption data for quarters 1–3 of 2017 were categorised by industry sector rather than DISCOs. These incompleteness and inconsistency with the data reflect our arguments underpinning the innovation of this paper and contribution as discussed in (Sect. 1) as well as a core rationale for the role and application of SNLs.

Nigeria currently has an installed electrical power generation capacity of about 12,522 MW, but only about 4000 MW is transmitted (NERC, 2021; USAID, 2021). Thus, Nigeria has not met its target of 23 GW by 2020. Moreover, the power generated is not optimally transmitted, and the available transmitted power also fluctuates significantly over an estimated 2000 MW range. This accounts for power rationing or variability in allocation to different areas and at different times. Certainly, the current generation pattern does not reach the targets set for sustainable energy by 2020. Based on Figs. 1 and 2, the distribution of electricity by some DISCOs which include Jos, Ibadan and Benin, covers more than one geographical zone. However, the Ikeja DISCO and Yola DISCO have the highest and lowest energy consumption values respectively. The former has a coverage limited to Lagos State within the South West zone while the latter has a coverage across 4 States within the North East zone. These States include Borno, Yobe, Adamawa and Taraba. Furthermore, as shown in Fig. 3 there is a significantly low concentration of medium voltage transmission lines within the aforementioned states and a high concentration of medium voltage transmission lines within Lagos State in the South West in general. Both Ikeja and Eko DISCOS supply energy limited to Lagos State, which highlights significant energy consumption and electricity availability in this State alone, the highest of any State in Nigeria.

Other data used to map the pattern of electricity access include that on, population,poverty, and household electricity consumption spending, sourced from the Nigerian National Bureau of Statistics (NBS) Oxford poverty and human development initiative (Alkire et al., 2015, 2020), and the United Nations Office for the Coordination of Humanitarian Affairs (OCHA).We discuss the application of this data in more details in Sect. 3.3.

### Satellite Night Lights (SNLs)

All SNLs data used in this work is retrieved by the Visible Infrared Imaging Radiometer Suite (VIIRS) on the Suomi National Polar-orbiting Partnership weather satellite system operated by the United States National Oceanic and Atmospheric Administration (NOAA) and the resulting data is archived by the National Aeronautics and Space Administration (NASA). To retrieve SNLs over Nigeria, the Day/Night band (DNB) was chosen, which is a panchromatic channel covering the wavelengths from 500 to 900 nm. This band is particularly sensitive to low-level radiation observed at night. The spatial resolution of this data is 750 m at nadir with pixel size increasing by the distance to the satellite’s nadir. Nevertheless, DNB consists of a nearly constant horizontal spatial resolution across the swath (NASA, 2017).

Before downloading the data from NASA’s data archive, we selected those days during the dry season in Nigeria, when at least ~ 90% of the country’s area were cloud-free, so that the signal would be unaffected by these atmospheric disturbances and the night lights on the ground would be visible. In total seven scenes were identified, which in some cases needed to be composed to either two or four separate images in order to cover the entire country for one night.

**Fig. 5:**
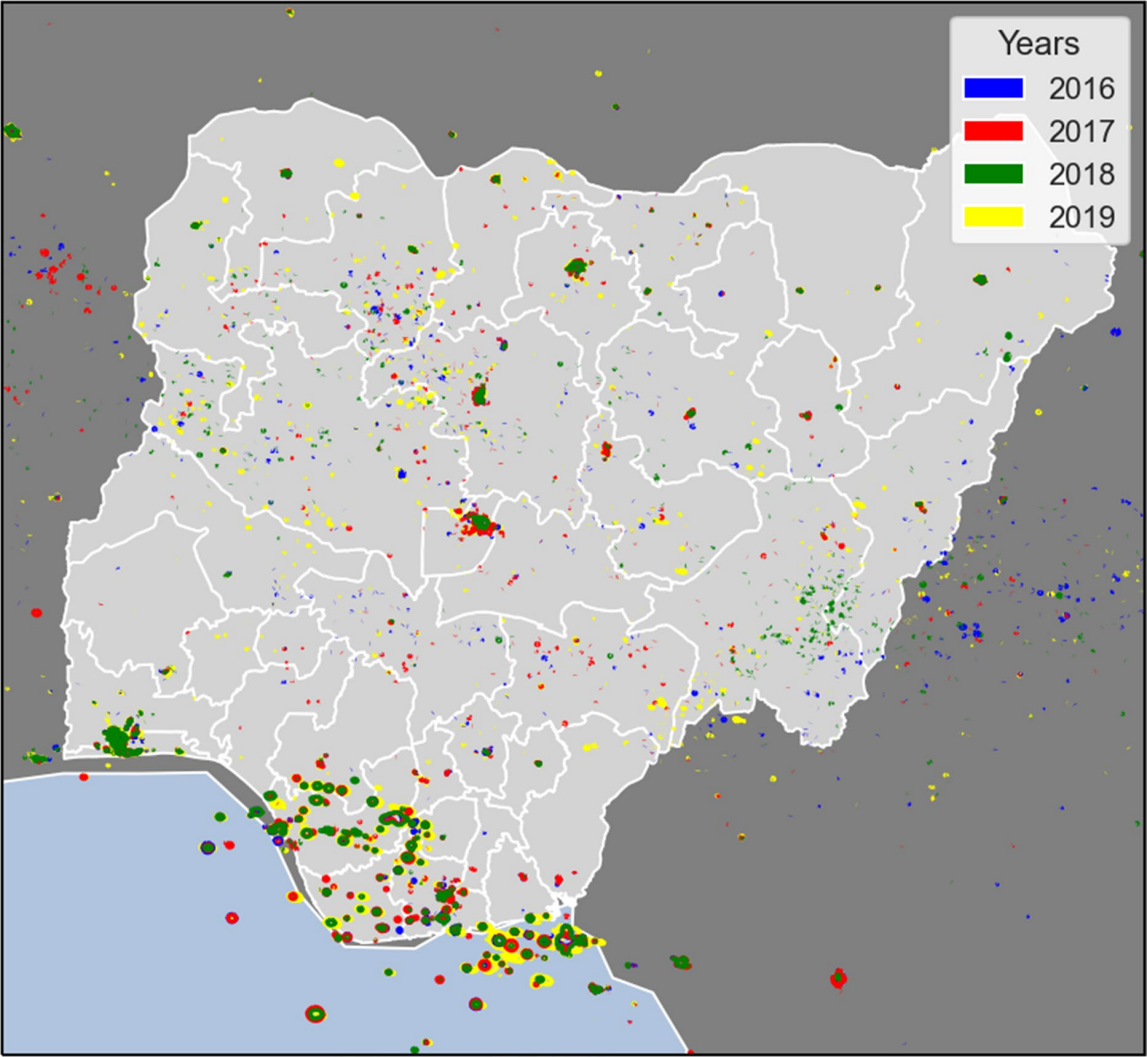
Detected satellite night lights (SNLs) in Nigeria 2016–2019

The data was processed using Python, where the NetCDF files (DNB observations and geolocation-data-product) were read and information at each pixel location was extracted. The radiance units are in Watts/cm^2^/steradian and we applied a threshold from 0.3 to 2.8 × (10)^−8^ Watts/cm^2^/steradian which corresponds to the lower part of the instruments dynamic range of approximately seven orders of magnitude, from 0.3 × (10)^−8^ to 0.02 Watts/cm^2^/steradian. Note that the SNLs images have been sorted according to the start of their winter season. An image captured in January 2018, for example, is therefore part of the winter season of 2017, which includes the months December 2017, January and February 2018. Individual images are described by date and time stamps of VIIRS/NPP Day/Night Band 6-Min L1B Swath SDR 750 m NRT (NASA, 2017). The last column in Table 1, describes the weather conditions which influence the image quality. A time series of the data reported in Table 1 is illustrated in Fig. 5.

**Table 1:** SNLs and satellite images for Nigeria in Winter-Season

Winter season	Image date	Image time	Image quality
2017	22 December 2016	1:06 UTC	Slightly influenced by atmospheric moisture
		1:12 UTC	
2018	27 December 2017	0:30 UTC	Clear
2018	18 January 2019	1:12 UTC	Southern Regions influenced by semi-transparent clouds
		1:18 UTC	
2018	31 January 2019	0:30 UTC	Clear
2018	17 February 2019	0:12 UTC	South South region mostly influenced by atmospheric moisture
		1:48 UTC	
		1:54 UTC	
2019	16 January 2020	0:18 UTC	Large portions of the country are influenced by moisture
		01:54 UTC	
		02:00 UTC	
2019	16 March 2020	0:42 UTC	Clear

*Image date and time stamps on VIIRS/NPP Day/Night Band 6-Min L1B Swath SDR 750 m NRT. Data Source (NASA, 2017)

### Integration of ground and SNLs data to develop insights on sustainable development indicators

To develop insight on electricity access in Nigeria, we select data for all the Nigerian states that can help reveal patterns about the affordability, accessibility, and adequacy of electricity in Nigeria when merged with SNLs data. We focus on key sustainable development indicators including population density, multiple poverty and expenditure on energy and fuel, electricity access, and electricity consumption. For each indicator considered, we optimise the related data by appropriately tagging missing values, ensure consistent naming and labelling, and units of measurement etc. For instance, we use electricity consumption of DISCO zones, and household and population data of states to analyse electricity consumption per capita and households. We also calculated the cumulative figures for all states within the geographical and DISCO zones in Nigeria, using averages. Each data set is then restructured to a usable format that allows us to simply map them to the SNLs data. We then conduct a simple descriptive statistical analysis and map each data for the different sustainable development indicators with SNLs data to capture logical correlations and patterns.

For example, to compare the poverty data to SNLs, the mean radiance (*MR*) per geographical zone was calculated by evaluating each pixel’s location. If the location was found to be within the bounds of a specific region it was included in the averaging process. Only pixels with SNLs values between the applied threshold were counted.

Furthermore, the ratio between light pixels and dark pixels is introduced as:

1 $$Ratio= \frac{Number Pixel with Radiance>0}{Number Pixel with Radiance \le 0}$$

This ratio indicates how illuminated a region is overall: The larger the value, the brighter that region at night as seen from the satellite.

The third variable we introduce is the normalised mean radiance (*NMR*):

2 $$NMR=\frac{MR}{2\bullet max(MR)}$$

where the mean radiance *MR* is the average radiance over pixels found in a particular region and $max(MR)$ is the maximum *MR* found in the entire country. For example, Lagos has the highest *MR* of the entire country with 0.27 Watts/cm^2^/steradian. All other regions are adjusted to twice this maximum, in order to create a notionally common scale which is comparable to the Nigerian Multidimensional Poverty Gap Indices (MPI) described in Sect. 4.2.

## Discussions of results, summary of key outcomes and narratives emerging

In this section, we present and explain our results and outcomes. Figure 5 provides a visual approximation of the availability of electricity in Nigeria based on SNLs. The detected SNLs time series from 2016 to 2020 generally shows minimal changes in electricity supply or accessibility in the country. However, there are improvements which can be seen in terms of more radiance of electricity, for instance from 2017 to 2018 in the north-eastern and eastern part of the country, and in 2019, off the coast in the southern part of the country. However, the SNLs data underpins the dominant presence of electricity in the southern part of the country where over 90% of the power generating plants are located, as well as major cities in the north, including Abuja (FCT) and Kano and to a lesser extent Kaduna, which are marked on the map.

There are only eight states with mean radiances above the average radiance in Nigeria of 0.027 × 10^−8^ W/cm^2^ and, apart from Abuja (FCT), are all located within the South South, South West, and South East regions. It is not surprising that the highest mean night lights reading is in the Lagos area with approximately 0.2676 × 10^−8^ W/cm^2^. The World Bank report on poverty in Nigeria also notes a greater access to electricity in the southern part of Nigeria (World Bank, 2016). This SNLs data confirms that this trend has not changed significantly since 2016.

Another general trend is the concentration of electricity in established urban areas. This is largely limited to State capitals in the North while in the South there is a broader spread of night lights which suggests the disparity between urban settlements in the two regions. Urban and infrastructural development in general is higher in Southern Nigeria, despite the large differential in geographical land mass or surface area. This then leads to an intricate correlation with other demographic or socioeconomic indices such as population density and poverty. More generally, electricity is sparse in the Northern part of the country and concentrated more densely in the southern part and changes in electricity supply based on this data are minimal over the timeframe.

### SNLs and population

**Fig. 6:**
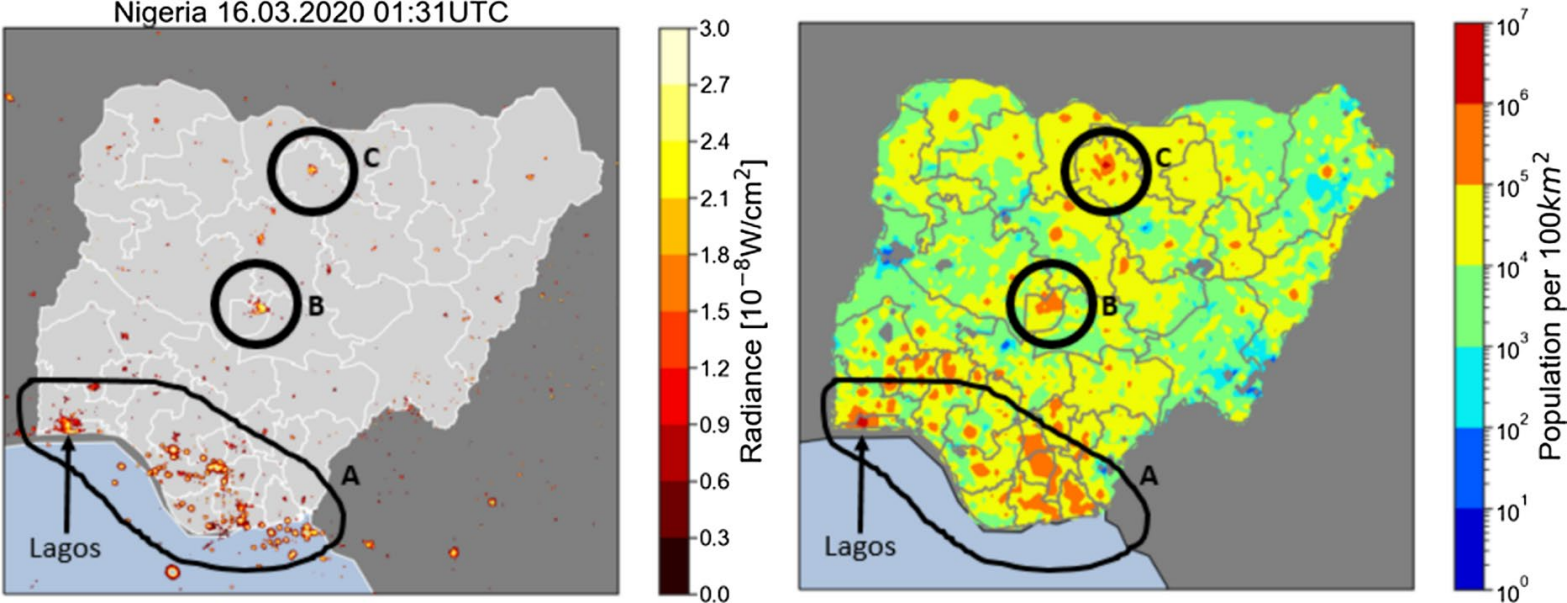
Left: SNLs map from 16/03/20. Right: Nigeria population density map

The SNLs records show the highest radiances for the areas in Nigeria with the higher population densities. These locations are also more urban areas and there is the clear indication that population density is higher in southern Nigeria. The points on the population density map with values from ${10}^{5}$ people per ${100 \mathrm{km}}^{2}$ correlate closely to the SNLs map (Fig. 6). The freeform shape marked A corresponds to the southern part of Nigeria where there is a higher radiance of detected night lights in the Figure to the left. The same area is marked in the population density map on the right and there is a strong correlation between the night lights and the population density point in the southern part of Nigeria with values of ${10}^{5}$ or more, depicted in the brown or darker points. The locations in the Northern part of the country, marked B and C, representing Abuja (FCT) and Kano respectively, which have the highest radiances are also depicted on the population map as the locations with the highest population density in northern Nigeria. Furthermore, the largest presence of electricity, marked by the radiance for a singular point in the country for the satellite detected lights is observed in Lagos, within the South West part of the country, the westernmost section in freeform A. This same location is observed to have the highest population density on the population map.

### SNLs and poverty

We find that there is evidence of chronic poverty in the North, with poverty rates decreasing more slowly than other zones of the country. Thus reinforces the spread of detected SNLs time series map (see Fig. 5), which highlights a significant disparity in the electricity radiance as well as spread or area of coverage between the North and South regions in Nigeria. Here, we use the Nigerian Multidimensional Poverty Gap Indices (MPI)^6^ for 2019 and the SNLs data for 2019 to analyse the relationship between electricity and poverty in Nigeria.

**Fig. 7:**
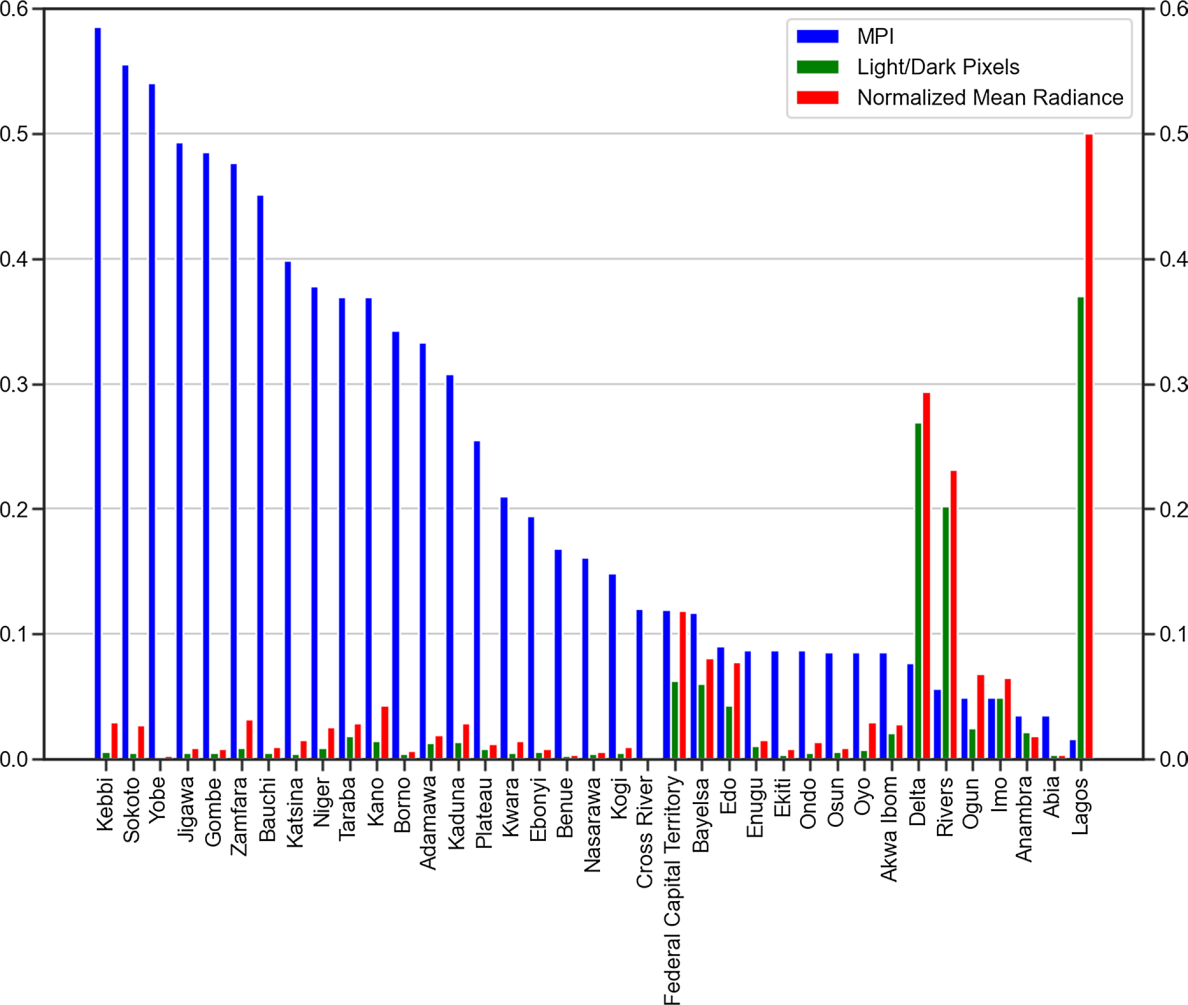
SNLs shown by normalised mean radiance values (unit: 10^−8^ W/cm^2^) and MPI for the Nigerian states

**Fig. 8:**
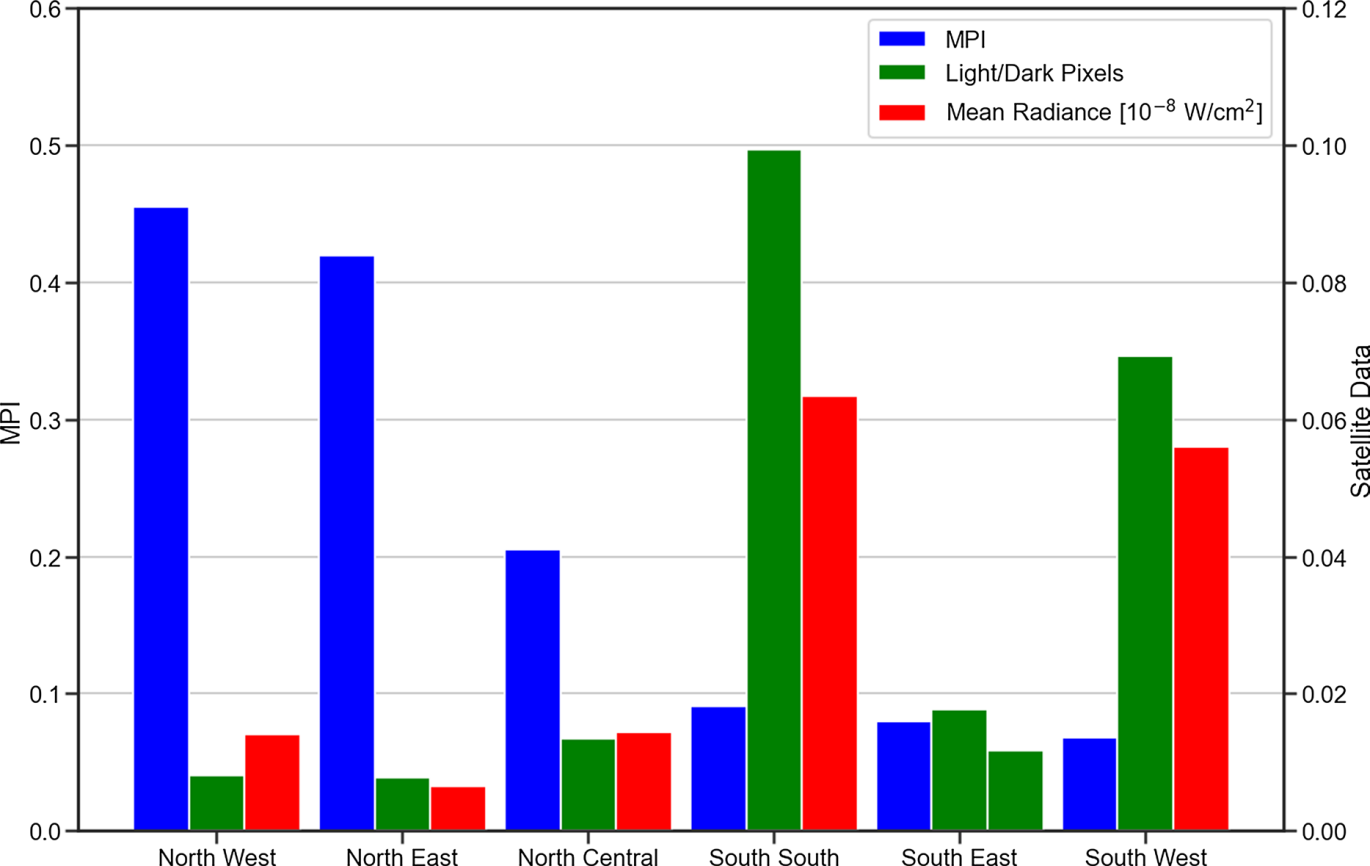
Average SNLs and normalised mean radiances for the six Nigerian geographical zones

As seen in Fig. 7, in general, the states with the lowest MPI, are the rich states like Lagos, Delta, and Rivers, which also have the highest normalised mean radiances at 0.5, 0.29, 0.23 radiance units respectively. There is a wide disparity of over 60% in the poverty headcount rate between Lagos State (South West) and Yobe State (North East), which has the lowest poverty headcount rate among the three States within the Yola DISCO. This reflects an inverse correlation across the states, validating the strong poverty-energy relationship, similarly for some states like Abia, Osun, and Anambra, which seem to be rich but with limited evidence light. This trend depicts the MPI analysis with all geographical zones showing an inverse relation besides the South East zone where the aforementioned states are located (Fig. 8).

**Fig. 9:**
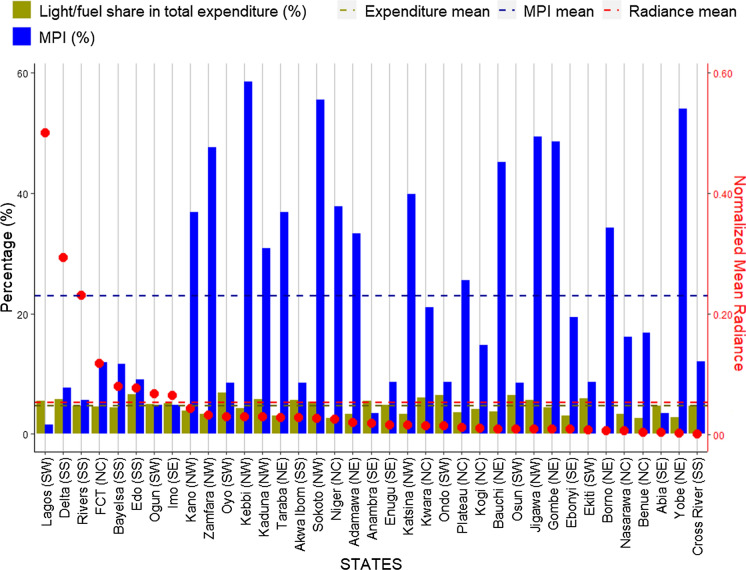
SNLs mapped the share of total expenditure spent on electricity/fuel for the states. *Borno state is not included in the expenditure survey

In Nigeria, the rich states spend a higher share of total expenditure on electricity. It is a mixed bag, however, in relation to electricity radiance and poverty presented in (Fig. 9). Of the eight states with highest radiance, (see Sect. 3.2), six spend above the average total expenditure on electricity (4.7%), with Edo state having the highest share of spend at over 6.6%, followed by Delta and Lagos at 5.8% and 5.5% respectively. The highest share of spend on electricity is observed within the rich (low MPI) yet low radiance states of Oyo at 6.9%, Ondo and Osun at 6.4% each, Kwara and Ekiti at 6.1% and 5.9% respectively. This trend may be attributed to the level of electricity distribution and consumption as most of states with highest spend on electricity are also located within the Ibadan, Benin, and Enugu DISCOs, ranking 2nd, 3rd, and 6th respectively.

**Fig. 10:**
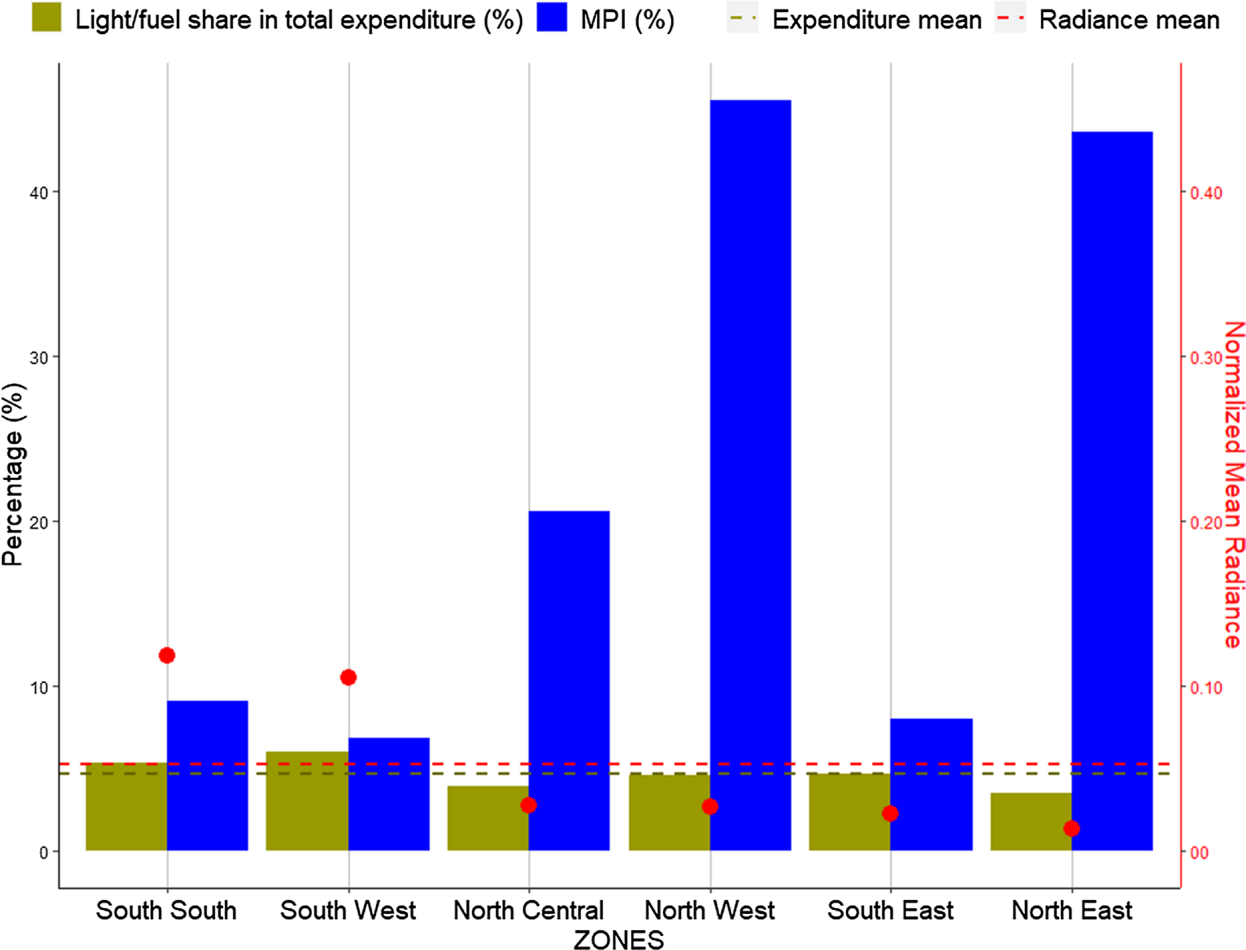
SNLs mapped the share of total expenditure spent on electricity/fuel for the Nigerian zones

Overall, twenty-one states (58%), excluding Borno, show a direct relation between spending on electricity/fuel share and radiance (electricity spread)—fifteen states spend lower with low radiance and six spending above average with high radiance. Nineteen states (53%) spend more (above average) than others on electricity, sixteen are rich states all located in the southern regions, and all but five (cited above) of them experience low electricity spread (radiance) showing a 38% inverse relation. This trend is reflected in the zonal analysis (Fig. 10), where the South South and South West regions spend the highest on electricity with high radiance. The South East and North West are sitting just below the average spend level and showing low radiance along with other low spending regions. This result is further contextualised with further analysis that links to household electricity access and dependency on electricity from the national grid versus other sources.

### SNLs and electricity sources, expenditure and access

#### SNLs and electricity sources

**Fig. 11:**
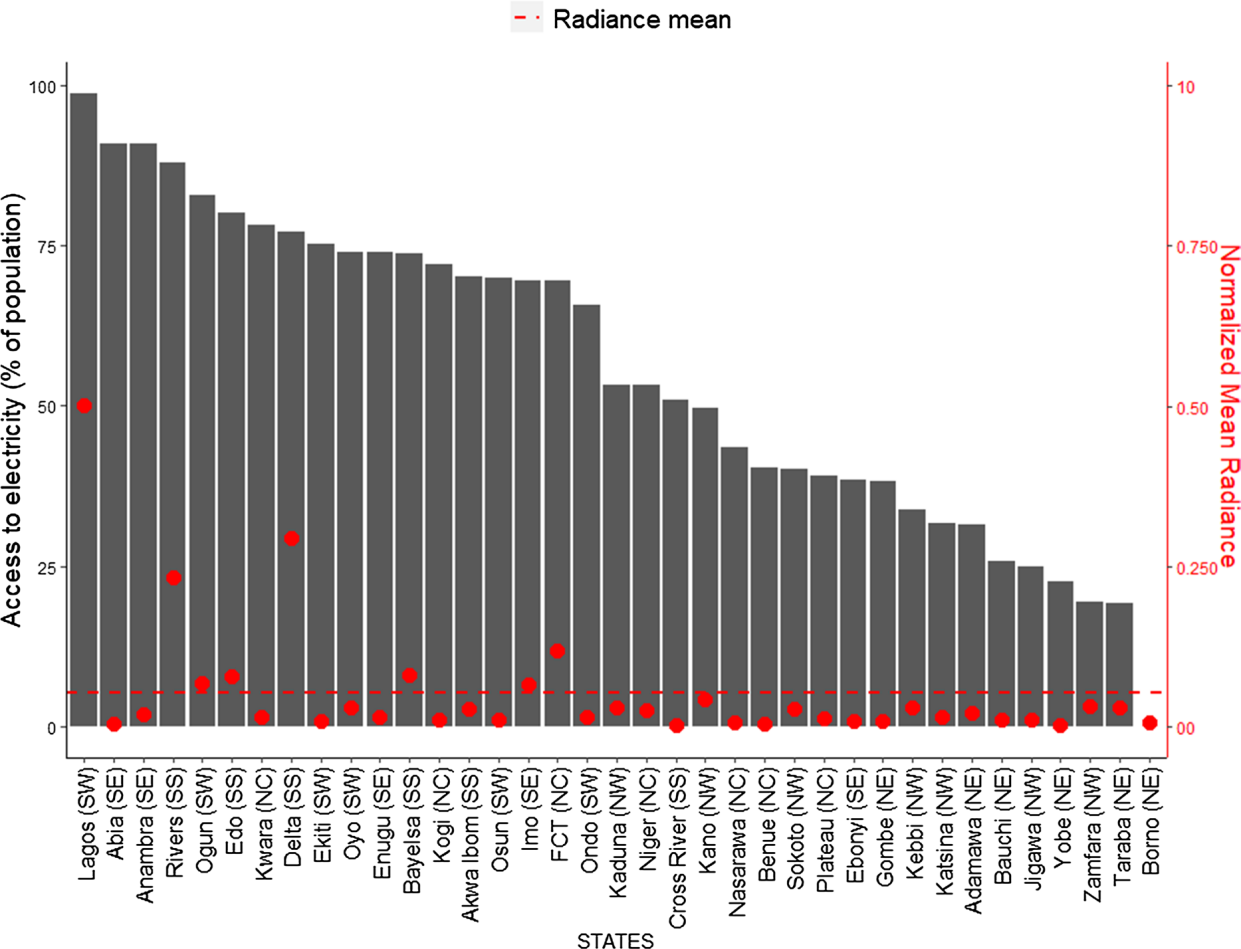
Access to electricity from any source for the Nigerian states

**Fig. 12:**
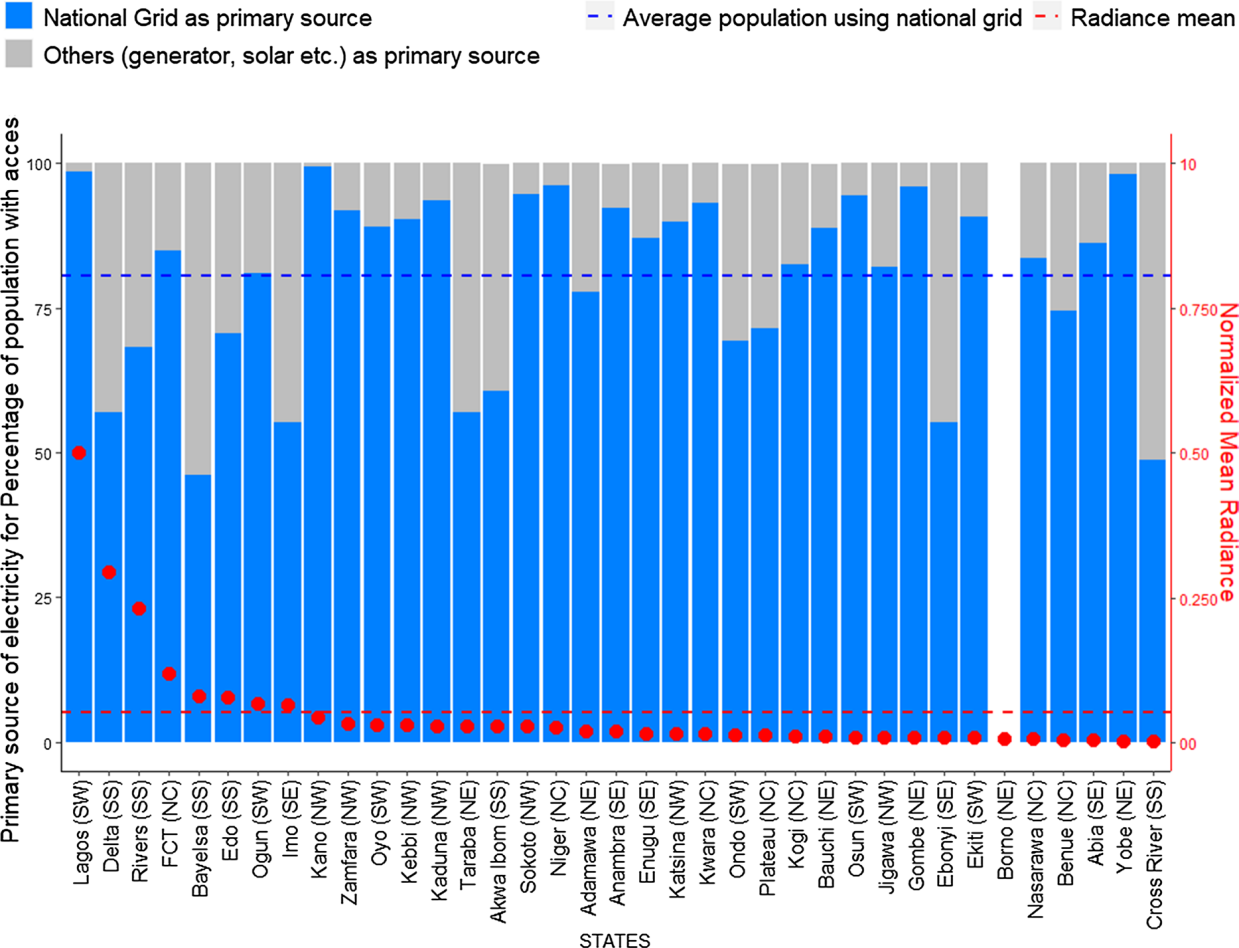
Primary source of electricity for households with access to electricity in Nigeria

At 64%, Nigeria has not met its target of increasing access to electricity from all sources to at least 75% by 2020. However, aggregate access to electricity has, improved from the 40% level of 2015 (NBS, 2020). There is also a great disparity in terms of overall access between the Nigerian states: over 98% of households in Lagos have access to electricity and the lowest is Taraba state, with only 19.2% of households having access to electricity. Thus, reemphasising our finding on the energy imbalance between the southern and northern regions of the country. We find that of the twenty-one states having at least 50% of households with access to electricity (Fig. 11), only eight have high radiance (above average) and include Abuja (FCT), Imo, Bayelsa, Delta, Edo, Ogun, Rivers, and Lagos states. These states also constitute those spending highest on electricity. Essentially, we that find that while there is access to electricity in some states, and households are spending a substantial amount on it, as  electricity spread is still low in general. When we further examined the dependency on grid electricity, we find that Kano, with its urban concentration of radiance sitting just below the average point, has the highest use of the national grid as the main source of electricity. Followed by Lagos, Yobe, Niger, and Gombe (see Fig. 12).

**Fig. 13:**
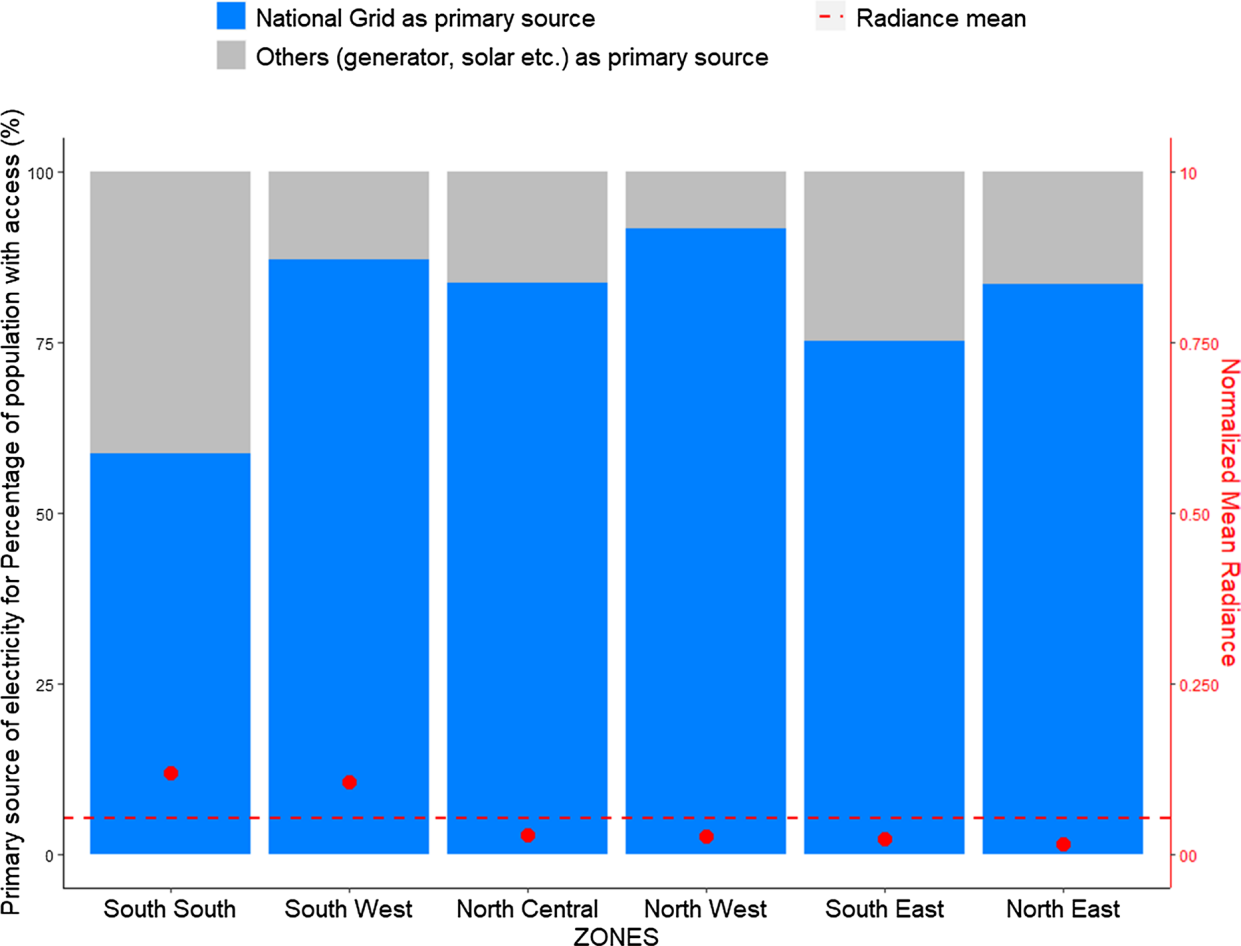
Primary source of electricity for households with access to electricity in the different regional zones of Nigeria

The average household dependency on electricity from the national grid is around 80.5% with most states above this point, but Lagos, Abuja (FCT), and Ogun states, have low radiance. Based on the thirty-four states with more than 50% household dependency on national grid only, seven have radiance above the average. This indicates an inverse relation between the use of national grid electricity and electricity spread in the states. This validates studies that indicate high dependencies on fuel powered generators to provide electricity in Nigeria (see Isola & Mesagan, 2017; World Bank, 2014), which are sources of electricity generation that are generally switched off for the night. As seen in Fig. 13, the result is reflected in the geographical analysis where the North West states depend most on the national grid for electricity with low output. And only the South West has high access to electricity and use of national grid as primary source and a high radiance.

#### SNLs and electricity consumption

**Fig. 14:**
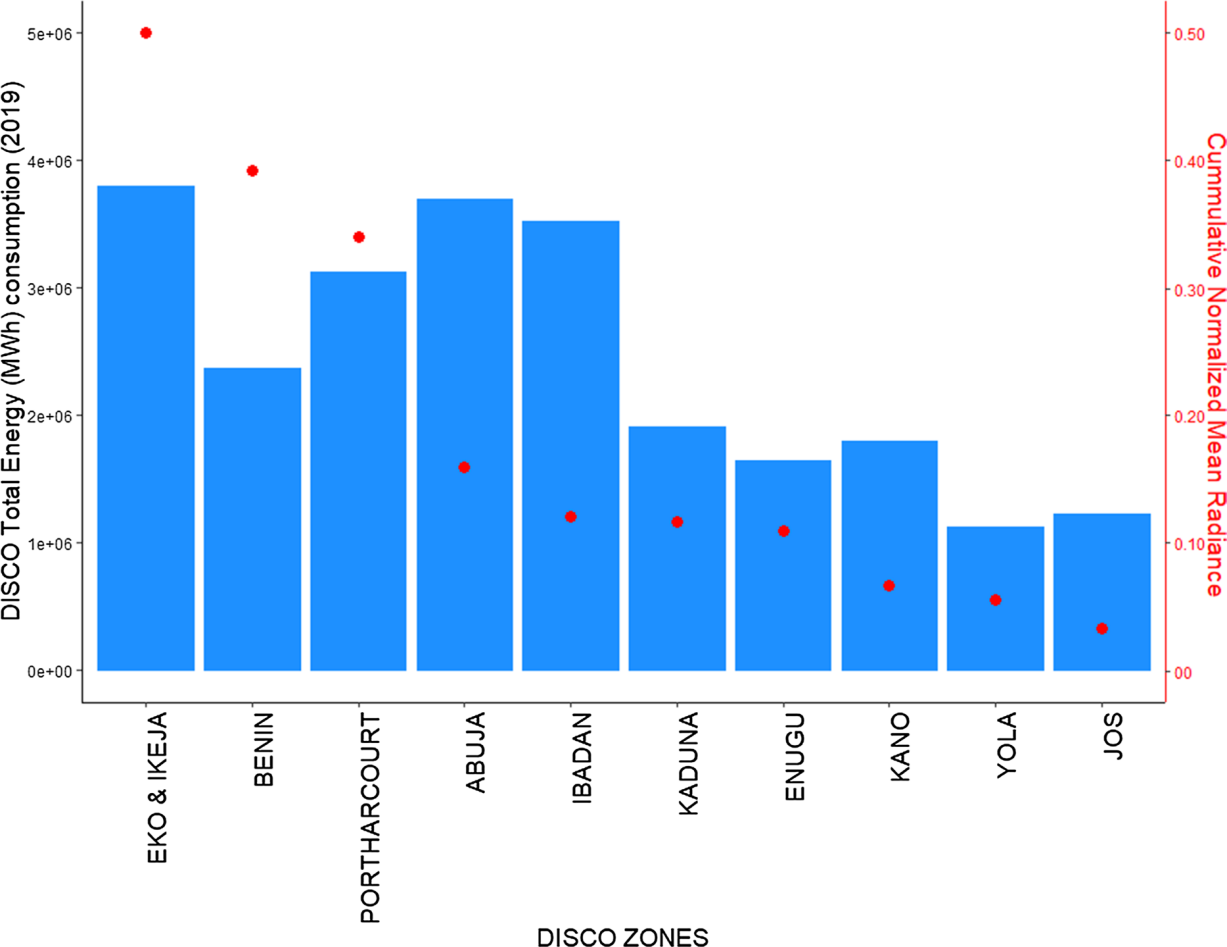
Total electricity consumption per DISCO zones and the relative cumulative radiance

**Fig. 15:**
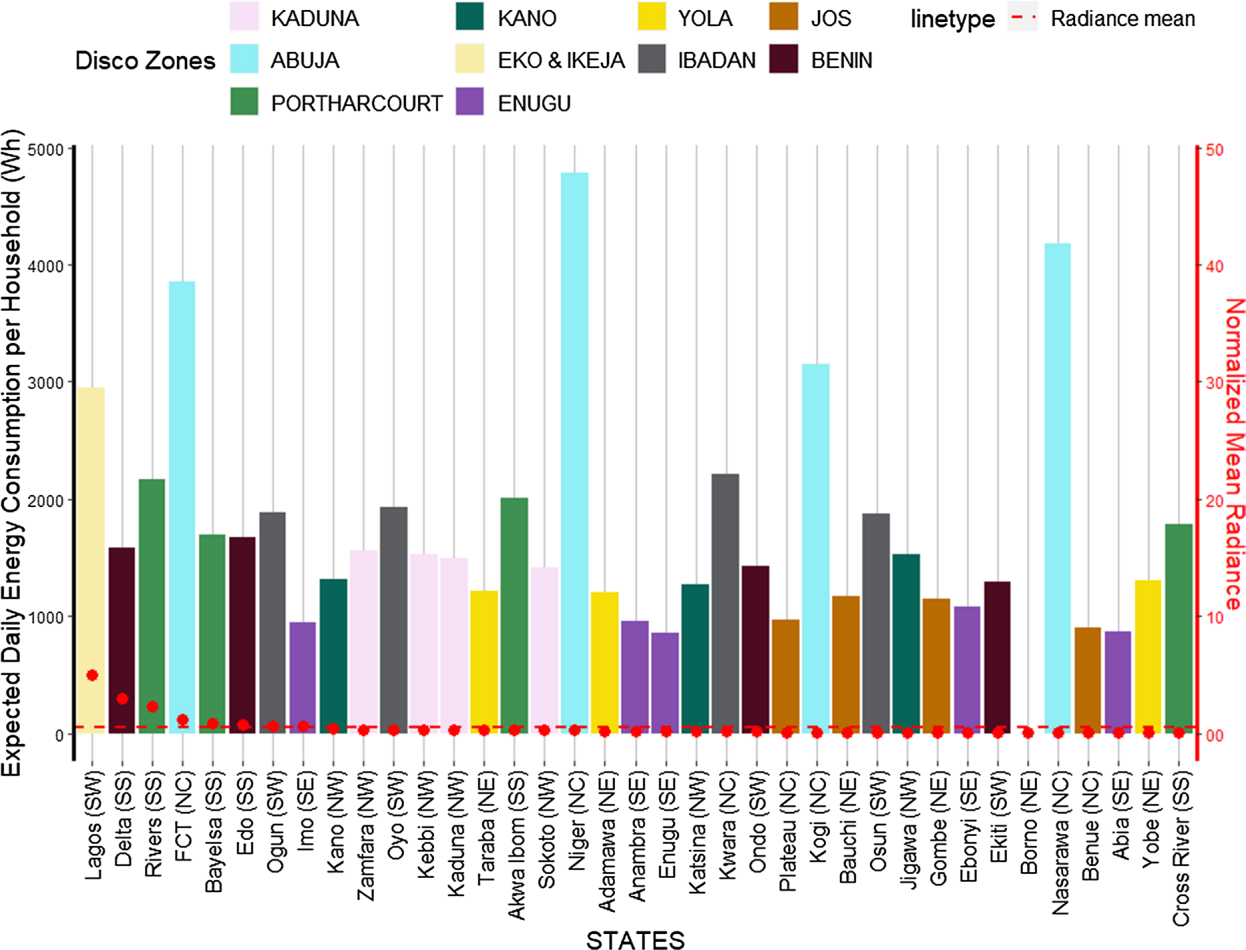
Electricity consumption per household for the Nigerian states

As seen in Fig. 4 (Sect. 3.1), the Ikeja, Abuja (FCT), Eko, Ibadan, Port Harcourt, and Benin DISCO zones supply and consume the most electricity respectively and show a direct relation to the cumulative electricity radiance spread in these zones (Fig. 14). Furthermore, the states that have higher access to electricity than others are in general located within these DISCO zones, including Enugu (see Fig. 11). When we zoom in to analyse the electricity available to households (relative to household sizes) for each state we also find that, the states within the above DISCO zones have higher consumption per household (Fig. 14). We note that this finding may be biased, given that we have derived the average household sizes and population in each state from a mix of secondary data sources. It does, however, align with the trend of energy consumption in the country and shows a strong relationship to the state of energy supply. For instance, in Lagos only 3.15 average household size has a daily household energy consumption of nearly 3000 Wh. While Jigawa state in the Kano Disco zone, which has the highest average household size of 8.15 (less than half the population of Lagos) has a daily household energy consumption of about 1500 Wh. An overview of the amount of electricity hours available during the day, provides a more nuanced, yet surprising perspective of how adequate the supply of electricity is to support daily activities  and livelihood (Fig. 15).

#### SNLs and electricity access

**Fig. 16:**
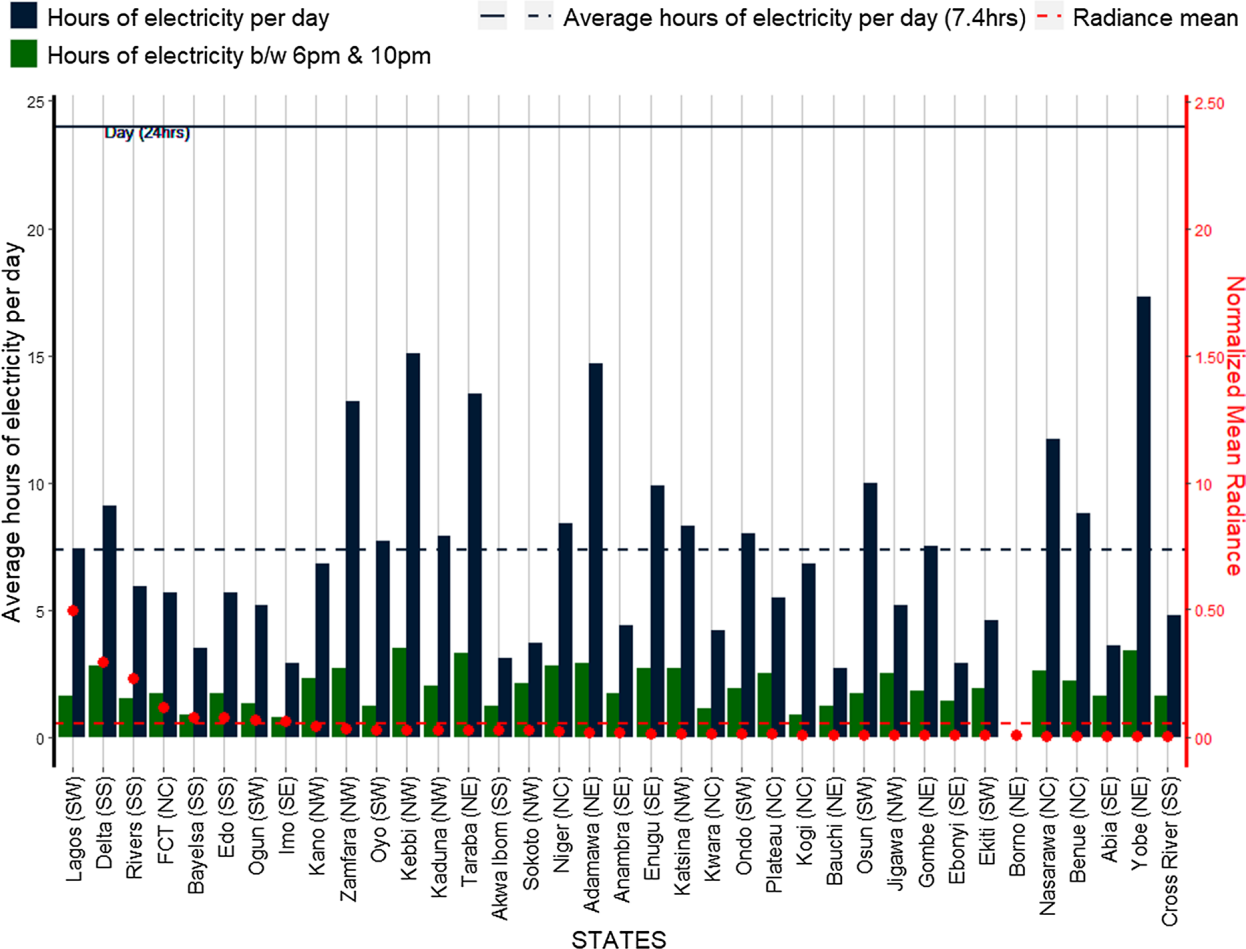
Average hours of electricity received per day by states. *Presented for night time periods of 6 pm and 10 pm with the mean radiances normalized to twice their maximum value for clearer representation

**Fig. 17:**
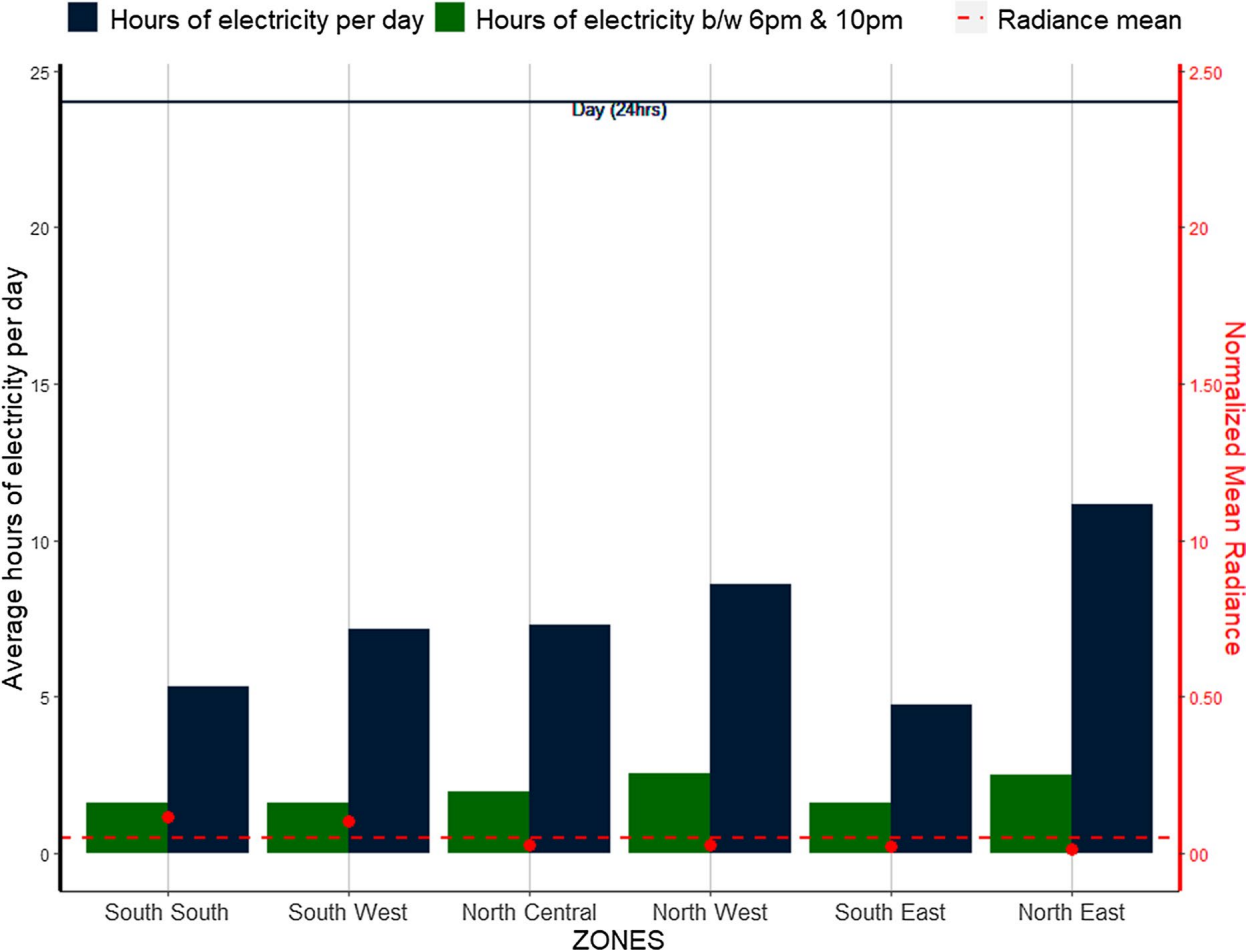
Average hours of electricity received per day by geo-political zones. *Presented for night time periods of 6 pm and 10 pm with the mean radiances normalized to twice their maximum value for clearer representation

In Fig. 16, we see that, on average the Nigerian population gets only 7 h of electricity supply per day (highlighting a gap of 17 h) to support living, livelihood, and daily activities for the whole of the country. Yobe state has the lowest gap, and as such highest number of electricity hours per day surpassing high radiance states like Lagos and Delta. This is an unexpected finding, given the very low radiance in the state and may be driven by the state’s dependence on national grid electricity (Fig. 12). Indeed, all but one (Delta state) of the high radiance states have no electricity supply for more than 7 h a day. It is also surprising that both Yobe and Lagos states (and Kano of course) are those with the highest degree of access and use of national grid electricity supply (Fig. 12), but with the lowest and highest radiance and electricity spread in the country respectively. This analysis can further validate the assertion of the high use of other sources (especially fuel powered generators) as a source of electricity. On the other hand, however, Yobe state is one of the North East states that show some degree of change in radiance between 2017 and 2018. This trend is maintained within the night time period of 6 pm to 10 pm where all the high radiance states (apart from Delta yet again) experience more than three hours without electricity supply along with other medium and low radiance states (Fig. 16). Yobe and Kebbi states in the North East and North West regions respectively show just over an hour on average without electric power supply during the night. Indeed, states in the North East and North West have the highest electricity hours per day and night in Nigeria, while also having some of the lowest radiance and electricity spread (Fig. 17).

## Conclusions and recommendations

In this paper, we set out to investigate progress around access to electricity and bridging the gap between disproportionate spread of electricity across the geographical regions in Nigeria using SNLs. The results and findings emerging suggest that minimal progress has been made and the challenge of disproportionate spread of electricity persists across the country with most of the electricity visibility concentrated in the Southern regions, state capitals and industrial regions.

Crucially, we find that the present pathway to the nation’s sustainable energy development agenda is focussed more on creating additional or new power generation capacity to accelerate and bridge the gap of insufficient supply and the disproportionate spread of electricity, with limited consideration or prioritisation of societal wellbeing and development impacts and implications. In particular, the indirect, unintended and unanticipated consequences in relation to the different sustainable development indicators. There is a need for policy makers to carefully consider and effectively balance the different strategies and pathways around enabling and realising access to electricity in Nigeria, especially for the share of the population that are vulnerable and exposed to energy poverty. Moreover, there is a need for more policy intervention and incentives to ensure that the prolonged issue of insufficient access to electricity does not trigger and/or exacerbate other forms of poverty (such as fuel, food, and housing) due to increased reliance and in turn higher share of disposable income spending on self-generated sources (e.g., power generators). This has the potential to widen inequality gaps, and result in human and economic development exclusion as well as health challenges (i.e., poor air quality, due to increased pollution).

There are several directions for future research consideration. First there is the need for more in-depth qualitative and quantitative research to further explore the relationship between electricity supply and maintaining and/or sustaining living standards and livelihood in the country particularly in the areas (e.g., Northern regions) that are more vulnerable than others. One that will explore the phenomenon through primary data that is collected at the households level. Moreover, while we show how the various dimensions that define access to electricity relate to wellbeing and poverty, this does not indicate causation. More research will be needed to provide further insights to fully investigate the implications of access to electricity and/or lack of it on human and economic development. Thirdly, due to the unanticipated disruptions (economic and societal shocks) caused by the Covid-19 pandemic, there is the need to further investigate how this may or may not have set Nigeria further back on the trajectory of resolving the issues of unserved population without access to electricity and the disproportionate spread of electricity.

## Data Availability

All the data informing this study is publicly available. The data on population is available at https://nigerianstat.gov.ng/elibrary and high resolution population density maps are available at https://data.humdata.org/dataset/highresolutionpopulationdensitymaps-nga. Household energy consumption and electricity access data is reported in the Nigerian living standard survey developed by the World Bank and the Nigerian National Bureau of Statistics (NBS) https://microdata.worldbank.org/index.php/catalog/3827. Poverty data is derived from Oxford Poverty and Human development initiative (OPHI) is available at https://ophi.org.uk/multidimensional-poverty-index/data-tables-do-files/. For the Satellite Night Lights (SNLs) and other satellite imagery data this is available at https://modaps.modaps.eosdis.nasa.gov/services/about/products/viirs-c1-nrt/VNP02DNB_nrt.html

## Footnotes

1 United Nations sustainable development Summit (2019). Available at https://www.un.org/sustainabledevelopment/blog/2019/09/un-sdg-summit-opens/

2 United Nations Sustainable Development Goal 7 : Ensure access to affordable, reliable, sustainable and modern energy.Available at https://www.un.org/sustainabledevelopment/energy/

3 Trend of total population of Sub Saharan Africa.Available at https://data.worldbank.org/indicator/SP.POP.TOTL?locations=ZG

4 Full report on Nigerian power sector recovery programme. Available at https://documents.worldbank.org/en/publication/documentsreports/documentdetail/115731517496257028/nigeria-powersector-recovery-program

5 Sustainable Energy for All in Nigeria agenda and plan. Available at https://nigeriase4all.gov.ng/about

6 MPI is a multidimensional measure of acute deprivations across a range of indicators that include health, education, and other aspects of living standards to complement monetary poverty (Alkire et al., 2020; National Bureau of Statistics, 2020).
